# Role of 5-methylcytosine in determining the prognosis, tumor microenvironment, and applicability of precision medicine in patients with hepatocellular carcinoma

**DOI:** 10.3389/fgene.2022.984033

**Published:** 2022-09-16

**Authors:** Mingyuan Luan, Min Zhao, Haiying Wang, Rongjian Xu, Jinzhen Cai

**Affiliations:** ^1^ Qingdao University Medical College, Qingdao, Shandong, China; ^2^ Center of Laboratory Medicine, Qilu Hospital of Shandong University (Qingdao), Qingdao, Shandong, China; ^3^ Key Laboratory of Sustainable Development of Marine Fisheries, Ministry of Agriculture and Rural Affairs, Shandong Provincial Key Laboratory of Fishery Resources and Ecological Environment, Yellow Sea Fisheries Research Institute, Chinese Academy of Fishery Sciences, Qingdao, China; ^4^ Department of Thoracic Surgery, The Affiliated Hospital of Qingdao University, Qingdao, China; ^5^ Organ Transplantation Center, The Affiliated Hospital of Qingdao University, Qingdao, Shandong, China

**Keywords:** 5 mC methylation, hepatocellular carcinoma (HCC), immune escape, immunotherapy, biomarker

## Abstract

**Background:** 5-methylcytosine has a profound impact on the development and progression of hepatocellular carcinoma. The aim of this study was to investigate the usefulness of 5-methylcytosine in determining the prognosis, tumor microenvironment, and applicability of precision medicine in hepatocellular carcinoma.

**Methods:** We collected data of seven hepatocellular carcinoma cohorts (The *Cancer* Genome Atlas, International *Cancer* Genome Consortium, GSE14520, GSE6764, GSE9843, GSE63898, GSE76427). An unsupervised clustering method was used to identify novel subtypes of hepatocellular carcinoma based on the expression 5-methylcytosine gene signatures. The 5-methylcytosine score was determined using the least absolute shrinkage and selection operator method based on the differential expression of genes in the identified subtypes. Subsequently, we investigated the association between 5-methylcytosine-based clusters (according to the 5-methylcytosine score) and clinical outcomes, immunophenotypes, classical molecular subtypes, and therapeutic opportunities in hepatocellular carcinoma. Finally, we examined the sensitivity of patients with high 5-methylcytosine score to drugs.

**Results:** We identified two hepatocellular carcinoma-specific, 5-methylcytosine-based subtypes (clusters 1 and 2). Cluster 1 exhibited significantly higher 5-methylcytosine scores versus cluster 2. The 5-methylcytosine-based subtypes accurately predicted classical molecular subtypes, immunophenotypes, prognosis, and therapeutic opportunities for patients with hepatocellular carcinoma. Cluster 1 (high 5-methylcytosine score) was characterized by lower anticancer immunity and worse prognosis versus cluster 2 (low 5-methylcytosine score). Moreover, cluster 1 (high 5-methylcytosine score) exhibited low sensitivity to cancer immunotherapy, but high sensitivity to radiotherapy and targeted therapy with lenvatinib.

**Conclusion:** The novel 5-methylcytosine-based subtypes (according to the 5-methylcytosine score) may reflect the prognosis, tumor microenvironment, and applicability of precision medicine in patients with hepatocellular carcinoma.

## Introduction

In 2018, hepatocellular carcinoma (HCC) ranked third among the leading causes of cancer-related death, accounting for >700,000 deaths worldwide (Bray et al., 2018). Despite rapid advancements in diagnostic and treatment strategies, 80% of patients are diagnosed with advanced disease, thus missing the optimal time for surgery (Kanwal and Singal 2019). Notwithstanding the merits of molecular targeting agents and immunotherapy, improvements in patient survival have been modest due to the high degree of heterogeneity in the tumor microenvironment (TME) of HCC (El-Khoueiry et al., 2017; Llovet et al., 2018).

The TME is a complex system which includes cancer cells, immune cells, and extracellular matrix (Chew et al., 2017). Due to TME heterogeneity, cancer cells of patients with the same pathological stage and grade may display distinct behaviors. This may result in varied clinical responses to the same treatment and impede the use of precision medicine (Burrell et al., 2013; Prasetyanti and Medema 2017).

Molecular subtype is a competent method which showed huge potential in addressing heterogeneity and determining the applicability of precision treatment in patients with HCC. A considerable amount of research has been conducted over the past decade to develop molecular subtype systems based on RNA sequence data. For example, classifications have been proposed by Boyault et al. (G1–G6) (Boyault et al., 2007), Chiang et al. (five subclasses) (Chiang et al., 2008), Hoshida et al. (S1–S3) (Hoshida et al., 2009), Désert et al. (four subclasses) (Désert et al., 2017), and Yang et al. (C1–C3) (Yang et al., 2020). Although the research groups developed their molecular subtype systems based on special criteria and algorithms, the long detection period and non-negligible diversity of each molecular subtype may impede their clinical application. Thus, a more rapid and accurate molecular subtype is required to promote the use of precision medicine.

In recent years, an increasing number of studies have focused on genome methylation, including 5-methylcytosine (5 mC), N6-methyladenosine, and N1-methyladenosine. Research has illuminated that the frequency and number of aberrant DNA methylations are closely associated with HCC(Nishida et al., 2008). A previous study demonstrated that 5 mC methylation plays a key role in the occurrence and development of HCC (Hlady et al., 2019). Villanueva et al. revealed that 5 mC methylation is closely associated with clinical stages, progression, prognosis, and survival rate in HCC(Villanueva et al., 2015). Moreover, in recent years, accumulating evidence has suggested that 5 mC shapes TME heterogeneity by affecting genomic stability, determining the state of cancer cell differentiation, and clarifying cell identity (Kandimalla et al., 2013; Bogdanović and Lister 2017; Kelly and Issa 2017; Biswas and Rao 2018). This evidence indicated that DNA methylation-based molecular subtypes may perform well in addressing the TME heterogeneity in HCC. Nevertheless, the high economic burden and complexity associated with methylation profiling are detrimental to the clinical application of DNA methylation-based molecular subtyping.

To address the aforementioned challenges, we developed a 5 mC-based subtyping system from the mRNA perspective. A prognosis-associated signature (5 mC score system) was subsequently developed and validated, which showed good performance compared with previously established signatures. We assessed the correlation among 5 mC subtypes (and 5 mC scores), TME heterogeneity, immune phenotypes, clinical characteristics, and therapeutic opportunities in HCC. Finally, a promising agent (irinotecan) was identified for the treatment of patients with a high 5 mC score, that may improve the current population-based therapeutic strategies for HCC.

## Methods

### Data collection and processing

We collected data from two RNA-sequencing and five microarray cohorts for analysis. Patients without detailed clinical information were eliminated. The RNA-sequencing cohorts included the Liver cancer—RIKEN, JP project (LIRI-JP) cohort (n = 231) and The *Cancer* Genome Atlas-Liver Hepatocellular Carcinoma (TCGA-LIHC) cohort (n = 373). The microarray cohorts included GSE14520 (n = 221), GSE6764 (n = 35), GSE9843 (n = 91), GSE63898 (n = 228), and GSE76427 (n = 115).

Gene expression data (raw counts) and clinical data of the LIRI-JP cohort were downloaded from the International *Cancer* Genome Consortium (ICGC) portal (https://dcc.icgc.org/projects/LIRI-JP). Raw counts were transformed into transcripts per million (TPM) values for subsequent analysis.

Gene expression data (raw counts), methylation data, copy number data, clinical data, and sample information of TCGA-LIHC cohort were downloaded from TCGA website (https://portal.gdc.cancer.gov/repository). Raw counts were transformed into TPM values for subsequent analysis. Mutation data of TCGA-LIHC cohort were obtained from Broad Institute’s GDAC Firehose (http://gdac.broadinstitute.org/). Differentially expressed genes (DEGs) between cancer and normal tissues were identified using the DESeq2 R package based on raw counts data (Love, Huber, and Anders 2014). Differentially expressed methylation sites between cancer and normal tissues were identified using the ChAMP R package (Tian et al., 2017).

The expression data and detailed clinical information of the GSE14520, GSE6764, GSE9843, GSE63898, and GSE76427 cohorts were downloaded from the Gene Expression Omnibus (GEO) (http://www.ncbi.nlm.nih.gov/geo/). Raw expression data of the GSE14520, GSE6764, GSE9843, and GSE63898 cohorts were normalized using the robust multi-array average method in the Affy R package. Raw expression data of the GSE76427 cohort were normalized using the robust spline normalization method in the lumi R package. The corresponding clinical information was obtained from the supplementary files of the articles to which they belonged. We merged the datasets downloaded from GEO website and composed the GEO-meta dataset.

All datasets were combined, and batch effects were eliminated by applying the “Combat” algorithm in the sva R packages. The merged data were examined by t-SNE algorithm.

Detailed information on these cohorts is provided in Supplementary Tables S1A–H.

### Unsupervised clustering of 12 5 mC regulators

The 5 mC regulators can be divided into three categories: writers, erasers, and readers. In this analysis, we collected 5 mC regulators from previous studies (Schübeler 2015; Wu and Zhang 2017; Ginder and Williams 2018; Chen et al., 2020; Hu et al., 2021a). Next, we selected the intersection of 5 mC regulators and the genes we had collected from our datasets. Finally, we selected 12 5 mC regulators, which included three writers (DNA methyltransferase three alpha [DNMT3A], DNMT3B, and DNMT1), one eraser (thymine DNA glycosylase [TDG]), and eight readers (methyl-CpG binding domain protein 1 [MBD1], MBD2, MBD3, MBD4, methyl-CpG binding protein 2 [MECP2], *n*th like DNA glycosylase 1 [NTHL1], single-strand-selective monofunctional uracil-DNA glycosylase 1 [SMUG1], and uracil DNA glycosylase [UNG]). We performed a consensus clustering analysis of the expression profiles of the 12 5 mC regulators using the ConsensuClusterPlus R package. To establish the real-world applicability of this classification system, we carried out unsupervised clustering for all cohorts after merging (n = 1,294) (Wilkerson and Hayes 2010). We set the parameters of the unsupervised clustering analysis as follows: pItem = 0.8; maxK = 6; reps = 100; clusterAlg = km; and distance = euclidean. Survival analysis for the identified clusters was carried out utilizing the “survival” and “survminer” R packages.

### DEG identification and functional annotation

We screened DEGs (criteria: |log fold change| >1 and adjusted *p*-value <0.05) between different 5 mC subtypes using the limma R package. To further explore the potential functions of 5 mC cluster-related DEGs, we carried out Gene Ontology and Kyoto Encyclopedia of Genes and Genomes (KEGG) analysis using the Database for Annotation, Visualization, and Integrated Discovery (DAVID) (Huang et al., 2009).

### Construction of the 5 mC score system

We developed a 5 mC scoring system to evaluate the 5 mC patterns of individual tumors. Firstly, we screened the DEGs using univariate Cox analysis. DEGs with *p*-values <0.05 were selected for further analysis. Next, we applied least absolute shrinkage and selection operator (LASSO) Cox regression analysis with 10-fold cross-validation in the “glmnet” R package to screen for optimal 5 mC subtype-related gene signatures in HCC (Friedman et al., 2010). The 5 mC score was calculated based on the relative expression of each screened signature and its associated Cox coefficient. The calculation formula was as follows:

$$5mC\;score=\overset{n}{\underset{i=1}{\sum }}\left({Coef}_{i}*{Expr}_{i}\right)$$

Coefi refers to the LASSO Cox coefficient of signature i. Expri is the expression of the gene in the signature for patient i. Patients were divided into high and low 5 mC risk score groups according to the median value. To further evaluate the ability of the 5 mC score for the prediction of prognosis, we performed univariate and multivariate Cox regression analyses with several important clinical features using data from TCGA, ICGC, GEO-meta cohorts, and all patients. To render the system clinically applicable, we used clinical characteristics and the 5 mC score to develop a predictive nomogram utilizing the rms R package. Calibration plots were used to evaluate the predictive performance of the nomogram. Decision curve analysis was carried out to estimate the suitability of the nomogram for clinical use according to the study conducted by Iasono et al. (Iasonos et al., 2008).

### Prediction of the classical molecular subtypes of HCC

We analyzed the association between 5 mC subtypes and five different transcriptome-based HCC classifications, namely those proposed by Boyault et al. (G1–G6) (Boyault et al., 2007), Chiang et al. (five subclasses) (Chiang et al., 2008), Hoshida et al. (S1–S3) (Hoshida et al., 2009), Désert et al. (four subclasses) (Désert et al., 2017), and Yang et al. (C1–C3) (Yang et al., 2020). We downloaded the signatures of these classical molecular subtypes and predicted the subtypes through nearest template prediction (NTP) analyses in the GenePattern website (https://cloud.genepattern.org/). We used receiver operating characteristic (ROC) curves to evaluate the performance of the 5 mC score in predicting classical molecular subtypes. A series of immunotherapy, target therapy, and radiotherapy signatures established by previous research were collected (Supplementary Table S4).

### Estimation of the immunological characteristics of TME in HCC

The TME is a complex and heterogeneous system which includes cancer cells, immune cells, extracellular matrix, and various immunomodulators.

Initially, we collected 163 immunomodulators (four major histocompatibility complexes, 35 immunostimulators, 35 immunoinhibitors, three interferons, 30 interleukins, and 22 other cytokines) from The *Cancer* Immunome Atlas (Charoentong et al., 2017). We compared differences in expression between the 5 mC-based subtypes and calculated the correlation between 5 mC scores and their expression levels. Chen et al. summarized the process of the anticancer immune response cancer immunity cycle theory (Chen and Mellman 2013). The cancer immunity cycle included seven steps: release of cancer cell antigens (Step 1), cancer antigen presentation (Step 2), priming and activation (Step 3), trafficking of immune cells to tumors (Step 4), infiltration of immune cells into tumors (Step 5), recognition of cancer cells by T cells (Step 6), and killing of cancer cells (Step 7). We assessed these seven steps using the Tracking Tumor Immunophenotype (TIP) website (http://biocc.hrbmu.edu.cn/TIP/) (Xu et al., 2018). In recent years, numerous algorithms were developed to assess the levels of immune cells in the TME based on bulk RNA-sequencing data. However, different algorithms may exhibit non-negligible diversity. To avoid the potential diversity, we estimated the immune cell infiltration in the TME using six independent algorithms: Cibersort; Cibersort-ABS; MCP-counter; quanTIseq; TIMER; and xCell utilizing the TIMER 2.0 website (http://timer.comp-genomics.org/) (Li et al., 2020). Next, we selected the effector genes of tumor-infiltrating immune cells identified in previous studies (Hu et al., 2021b). Subsequently, we collected gene signatures positively correlated with the clinical response to treatment with an anti-programmed cell death-ligand 1 (anti-PD-L1 agent; atezolizumab) (Mariathasan et al., 2018). Finally, we collected a series of positive, negative, and hyperprogression gene signatures of immune checkpoint blockade (ICB) therapy (Supplementary Table S4).

### Collection of therapy-specific signatures, therapy targets, and other functional pathways

Critical therapy-specific signatures, including noninflamed TME-related oncogenic pathways, signatures related to epidermal growth factor receptor (EGFR) targeted therapy, and signatures related to radiotherapy, were collected from a previous study (Hu et al., 2021a). We also collected the drug targets of sorafenib and lenvatinib from the DrugBank database to further analyze the potential ability of the 5 mC score and 5 mC-based subtypes to predict therapeutic opportunities for patients with HCC. The hallmark pathways and KEGG pathways were collected from the MsigDB database (Liberzon et al., 2015).

### Screening for potential therapeutic agents

Expression profile data for a human cancer cell lines (CCLs) were downloaded from the Broad Institute *Cancer* Cell Line Encyclopedia (CCLE) (Ghandi et al., 2019). Drug sensitivity data for CCLs were obtained from The *Cancer* Therapeutics Response Portal (CTRP) version 2.0 (https://portals.broadinstitute.org/ctrp) and PRISM Repurposing dataset (19Q4; https://depmap.org/portal/prism/). Both datasets provided area under the curve (AUC) values as a measure of drug sensitivity; lower AUC values denoted increased sensitivity to treatment. Compounds with >20% missing AUC values were excluded. Next, K-nearest neighbor imputation was applied to impute the missing AUC values of the remaining compounds. The expression profile data of the human CCLs in CTRP and PRISM were obtained from the CCLE project and used for further analysis.

### Statistical analysis

All statistical tests were performed using the R statistical software (version 3.6.1, R Core Team; R Foundation for Statistical Computing, Vienna, Austria). Comparison of a continuous variable in two or more groups was performed using the Wilcoxon rank-sum test or Kruskal–Wallis test. The correlation between two continuous variables was evaluated using Spearman’s rank-order correlation. The survival curves for each dataset were generated by Kaplan-Meier analysis, and statistically significant differences were determined using the log-rank test. All survival analysis, including Kaplan-Meier and Cox analyses, were carried out for patients with survival times and status. Patients without survival times and status were eliminated. The ROC curve was used to assess the specificity and sensitivity of the 5 mC scores, and the AUC was quantified using the pROC R package (Robin et al., 2011). All *p*-values were two-sided, and *p* < 0.05 denoted statistically significant differences.

## Results

### Landscape and multi-omics analysis of 5 mC regulators in HCC

**FIGURE 1:**
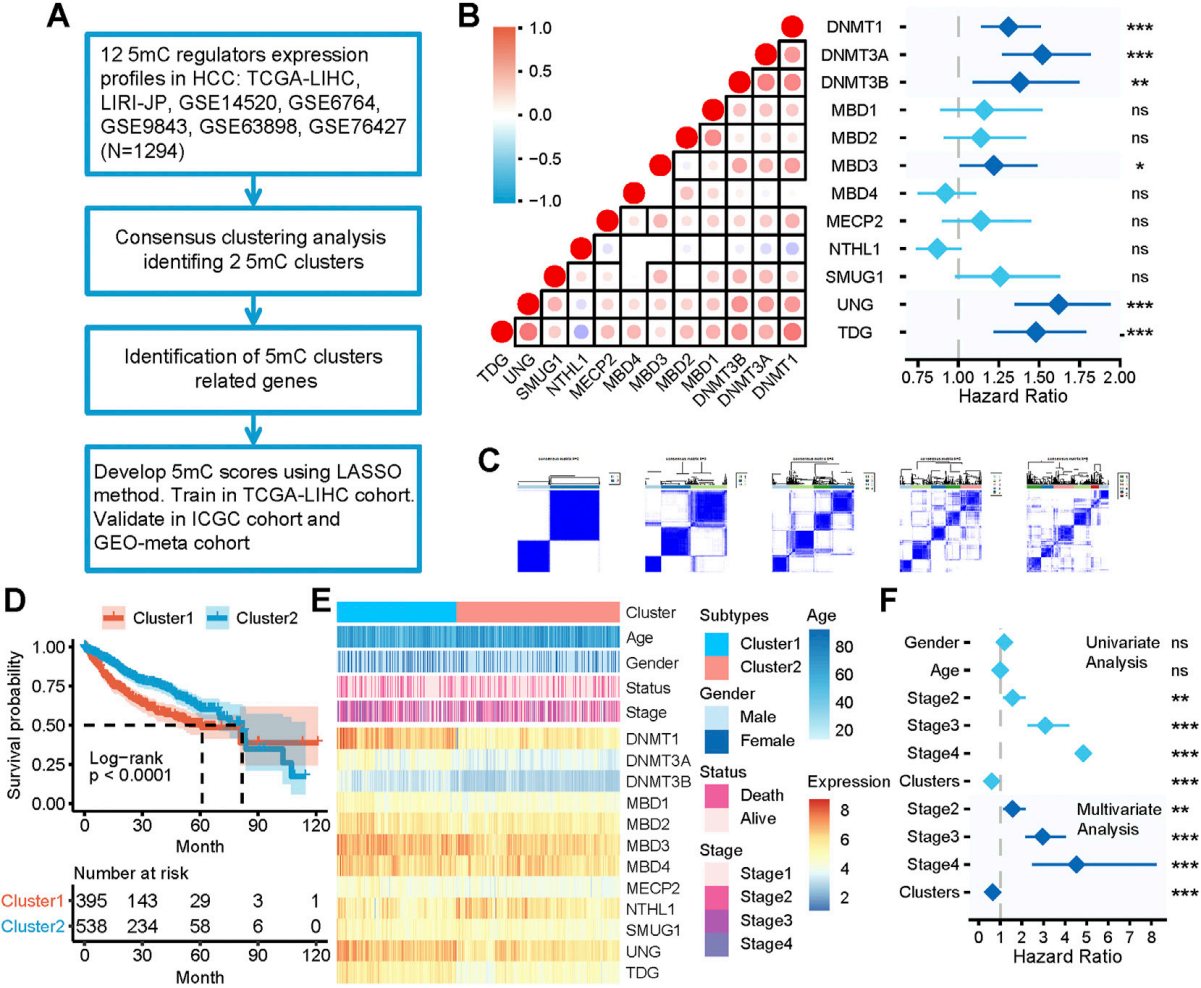
The clinical relevance of 5 mC clusters in the meta cohort. **(A)** Overview of developing 5 mC clusters and 5 mC scores. **(B)** The correlation among the expression of 5 mC regulators and the prognostic value. In the left panel, each point represents the correlation of the 5 mC scores and the scores of each step in the cancer immunity cycle. The points are color-coded based on the correlation. The red color indicates a positive correlation, whereas the blue color represents a negative correlation. The light color represents a low correlation and progressively darker colors represent a higher correlation. The black box represents a significant correlation (*p* < 0.05). In the right panel, 5 mC regulators were significantly correlated with patient survival and are colored in dark blue. The length of each line represents the range of the hazard ratio for each 5 mC regulator. **(C)** Consensus matrices of the meta cohort for k = 2–5. **(D)** Survival analysis of 5 mC clusters 1 and 2. **(E)** Association between 5 mC clusters, clinicopathologic characteristics, and 5 mC regulator expression. **(F)** Outcomes of univariate and multivariate Cox analyses of 5 mC clusters. The light blue color represents the outcome of univariate Cox analysis outcomes. The dark blue represents the outcomes of multivariate Cox analysis. The length of the line represents the range of the hazard ratio. ns, not significant, *p < 0.05, **p < 0.01, ***p < 0.001.

The 5 mC regulators exhibited a relatively low mutation rate in patients with HCC (Supplementary Figure S1A). Of the 372 patients with HCC in TCGA-LIHC cohort, 26 (6.99%) had mutations in 5 mC regulators. Among them, DNMT3A had the highest mutation frequency (2%), followed by TDG, MBD4, MECP2, UNG, DNMT1, DNMT3B, MBD1, MBD2, MBD3, NTHL1, and SMUG1. The copy number variation (CNV) of the 5 mC regulators in TCGA-LIHC cohort showed that DNMT3A, DNMT3B, and MECP2 exhibited widespread CNV amplification. DNMT1, MBD1, MBD2, MBD3, and NTHL1 showed CNV deletion (Supplementary Figure S1B). We further analyzed the correlation between CNV and the mRNA expression levels of the 5 mC regulators. We observed that the CNV of most 5 mC regulators was significantly positively correlated with the mRNA expression levels (Supplementary Figure S1B). We also analyzed the methylation status of the 5 mC regulators between HCC and normal tissues in TCGA-LIHC cohort. According to our results, most 5 mC regulators showed higher methylation levels (Supplementary Figure S1C). Moreover, the methylation showed a significant negative correlation with the expression of DNMT1, MBD2, and MBD3 (Supplementary Figure S1C). There were significant differences in the expression levels of 5 mC regulators between HCC and normal tissues in TCGA-LIHC cohort. We observed that DNMT1, DNMT3A, DNMT3B, MECP2, MBD1, and SMUG1 were significantly upregulated, whereas MBD4 was significantly downregulated, in HCC tissues compared with normal tissues (Supplementary Figure S1D). Most 5 mC regulators were significantly positively correlated with others. However, NTHL1 showed an opposite trend (Figure 1B). The Cox analysis showed that the majority of 5 mC regulators were adverse prognostic factors in all patients (Figure 1B).

### Identification of 5 mC subtypes in HCC

The workflow of developing 5 mC clusters and 5 mC scores in this study is illustrated in Figure 1A. As shown in Figure 1C, all patients were classified into several clusters based on the mRNA expression profile of 12 5 mC regulators using a consensus clustering algorithm. The findings showed that the entire cohort could be classified into two clusters, namely cluster 1 (n = 503) and cluster 2 (n = 791) (Supplementary Table S1B–H). The two clusters showed significant differences in prognostic outcomes (Figure 1D). Patients in cluster 1 showed significant worse survival outcomes compared with those in cluster 2. In addition, the clinicopathological characteristics and expression of 5 mC regulators showed different patterns between the two clusters (Figure 1E). Cluster 1 included younger patients, more females, patients with more advanced disease, and exhibited a higher mortality rate compared with cluster 2. Furthermore, univariate and multivariate Cox regression analyses revealed that 5 mC cluster was an independent prognostic factor (Figure 1F).

### Construction and validation of the prognostic 5 mC score system

**FIGURE 2:**
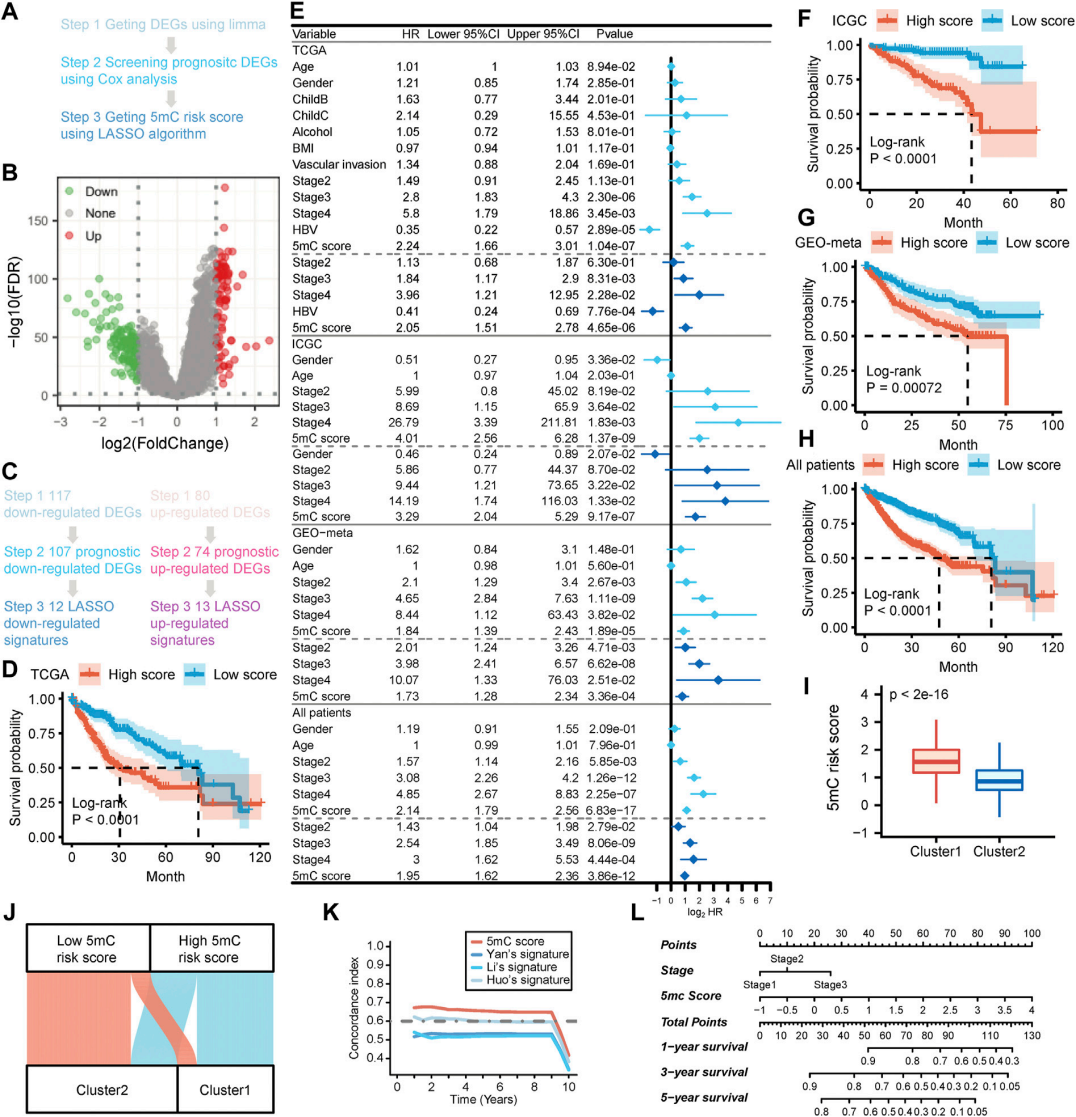
Developing the 5 mC gene signature, 5 mC score and exploring the clinical practice in the meta cohort. **(A)** The flow chart of the 5 mC score algorithm. **(B)** The volcano plot of the differentially-expressed genes (DEGs) between 5 mC subtypes. **(C)** Screening outcomes of 5 mC gene signatures using the LASSO algorithm. **(D)** Kaplan-Meier survival analysis of patients in the TCGA cohort. **(E)** Independent prognostic analysis of 5 mC scores in the TCGA, ICGC, GEO-meta cohorts, and all patients. **(F–H)** Kaplan-Meier survival analysis of patients in the ICGC and GEO-meta cohorts, and all patients. **(I)** Comparison of 5 mC scores between 5 mC clusters. **(J)** The distribution of 5 mC score level between clusters 1 and 2. **(K)** The c-index of 5 mC scores compared with other signatures. **(L)** Nomogram of 5 mC scores for clinical practice. *p < 0.05, **p < 0.01, ***p < 0.001.

The workflow adopted in this part of the study is illustrated in Figure 2A. Firstly, we identified 117 downregulated and 80 upregulated DEGs between the two 5 mC-based clusters in all patients (Figures 2B,C; Supplementary Table S2A). Next, a univariate Cox proportional risk regression model with a threshold of *p* < 0.05 was used to identify the DEGs associated with the overall survival of all patients. A total of 107 downregulated and 74 upregulated DEGs associated with survival were initially identified (Figure 2C; Supplementary Table S2A). Gene Ontology and KEGG enrichment analyses showed the DEGs were mainly enriched in metabolism and cell cycle (Supplementary Figure S1A, B). Thereafter, we calculated the 5 mC risk score using the LASSO method based on the survival information and expression profile of the survival-related DEGs. We trained the 5 mC score system using data from the TCGA-LIHC cohort. We also validated the system using data from the LIRI-JP and GEO-meta (GSE14520 and GSE76427) cohorts. The relative regression coefficients of survival-associated DEGs were subsequently calculated using a LASSO analysis. Coefficients of survival-associated DEGs were reduced to zero by forcing the sum of the absolute value of the regression coefficients to be below a fixed value. Using the LASSO method, a total of 25 survival-associated DEGs were selected as the most powerful prognostic markers (Figure 2C). Detailed information on these 25 survival-associated DEGs and their coefficients are presented in Supplementary Table S2A. The 5 mC score for each patient was calculated by combining the expression levels of the LASSO marker with the corresponding LASSO coefficients. Patients were classified into high- and low-risk groups based on the median value of their 5 mC scores. Kaplan–Meier survival analysis of data obtained from TCGA-LIHC cohort showed that patients in the high-score group were associated with a significant worse prognosis than those in the low-score group (Figure 2D). The univariate and multivariate Cox regression analyses showed that the 5 mC score was an independent predictive factor for patients in TCGA cohort (Figure 2E). Similar results were obtained in the analysis of the test set (LIRI-JP and GEO-meta cohorts) and all patients (Figures 2F–H). The univariate and multivariate Cox regression analyses also showed that the 5 mC score was an independent predictive factor for ICGC cohort, GEO meta cohort and all patients (Figure 2E). Our results showed that cluster 1 had significantly higher 5 mC scores than cluster 2 (Figure 2I). The 5 mC score could effectively quantify the 5 mC-based clusters (Figure 2J). To determine the biological meaning of the m5C score, we identified 61 upregulated differentially-expressed genes (DEGs) and 77 downregulated DEGs between the high and low 5 mC score groups (Supplementary Table S2C). Gene ontology and KEGG enrichment analyses showed the DEGs were mainly enriched in the metabolic process (Supplementary Figure S1C, D).

Previous studies identified several population-based prognostic signatures for patients with HCC, including those identified by Yan et al. (4 gene signatures) (Yan et al., 2019), Li et al. (3 gene signatures) (Li et al., 2017), and Huo et al. (10 gene signatures) (Huo et al., 2021). We analyzed the time-dependent c-index for 5 mC scores and these population-based signatures for all patients. The results revealed that 5 mC scores exhibited higher levels of the c-index than the three aforementioned population-based signatures in all patients (Figure 2K).

To improve the clinical application of 5 mC scores, we constructed a nomogram which incorporated the 5 mC score and disease stage (Figure 2L). Calibration plots showed that the nomogram could accurately predict survival at different time points (Supplementary Figure S2E). Decision curve analysis also showed that a comprehensive signature can provide better clinical benefit to patients in terms of prediction versus only the 5 mC score or only the disease stage (Supplementary Figure S2F–H).

### Association between clusters (according to the 5 mC score) and the TME in all cohorts

Most immunomodulators were upregulated in cluster 1 (Supplementary Table S3A). The expression of most immunomodulators was positively correlated with the 5 mC score (Supplementary Table S3B). The expression of C-C motif chemokine ligand 20 (CCL20), C-C motif chemokine receptor 6 (CCR6), and CCR10, which induce the recruitment of regulatory T (Treg) cells into the TME, was upregulated in cluster 1 and significant positively correlated with the 5 mC score. C-X-C motif chemokine ligand 14 (CXCL14), which can induce infiltration of M2 macrophages in the TME, showed significant negative correlation with the 5 mC score.

**FIGURE 3:**
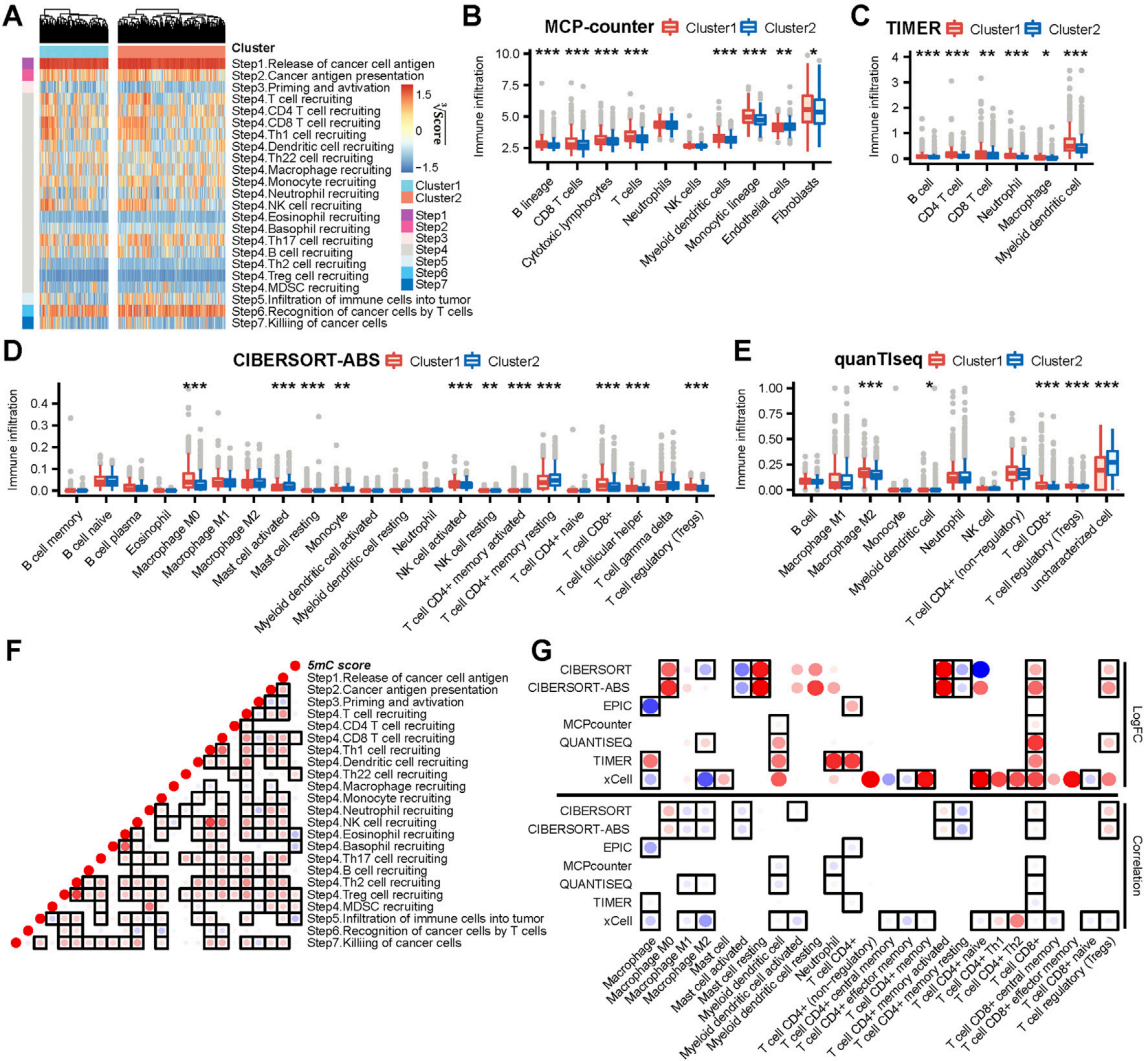
The 5 mC clusters and 5 mC score correlated with immune phenotypes in meta cohort. **(A)** The ssGSEA score heatmap of seven steps in cancer immunity cycles. **(B–E)** The differences in infiltration levels of immune cells between 5 mC clusters in the MCP-counter, TIMER, CIBERSORT-ABS, and quanTIseq algorithms. **(F)** The correlations between the 5 mC score and steps in cancer immunity cycles. Each point reports the correlation resulting from correlating 5 mC scores and ssGSEA scores of each step in the cancer immunity cycle. The points are color-coded based on correlation. The red color indicates a positive correlation, whereas the blue color represents a negative correlation. The light color represents a low correlation and the progressively darker color represents a higher correlation. The black box represents a significant correlation (*p* < 0.05). **(G)** The upper panel indicates that the logFC of the main type of immune cells between clusters 1 and 2. The bottom panel indicates that the correlation between the main type of immune cell and 5 mC scores. The darker red colors represent a higher logFC or a more positive correlation. The darker blue colors represent a lower logFC or a more negative correlation. The points are color-coded by the logFC (upper) or correlation (bottom). The red color indicates a positive logFC (upper) or correlation (bottom). The blue color represents a negative logFC (upper) or correlation (bottom). The light color represents a low absolute value of logFC (upper) or a correlation (bottom). In contrast, progressively darker colors represent a higher absolute value of logFC (upper) or a correlation (bottom). The black box represents a significant correlation (*p* < 0.05). *p < 0.05, **p < 0.01, ***p < 0.001.

*Cancer* immunity cycles are directly determined by the comprehensive performance of immunomodulators. Thus, we analyzed the activities of the cancer immunity cycle using the TIP website. We observed that many steps in the cycle, such as Step 1 (release of cancer cell antigen) and Step 4 (CD8^+^ T, T helper 22 [Th22], natural killer, and Th17 cell recruitment) (Figures 3A and Supplementary Figure S3A), were significantly activated in cluster 1. Eosinophil recruitment (Step 4), infiltration of immune cells into the tumor (Step 5) and recognition of cancer cells by T cells (Step 6) were significantly limited in cluster 1 (Figures 3A and Supplementary Figure S3A). Consequently, the activated steps of the cancer immunity cycle and immunomodulators may suggest higher infiltration of tumor-infiltrating immune cells in the TME in cluster 1. Thus, we used seven independent algorithms to assess the infiltration level of tumor-infiltrating immune cells in the TME. Most algorithms showed that, in cluster 1, there were high levels of infiltration of CD8^+^ T, myeloid dendritic, and Treg cells (Figures 3B–E,G; Supplementary Table S3B–D) compared with cluster 2. In contrast, endothelial cells showed significantly lower levels of infiltration in cluster 1 compared with cluster 2. These results implied that cluster 1 may be characterized by high levels of anti-tumor characteristics (CD8^+^ T cells, release of cancer cell antigen [Step 1], CD8^+^ T cell recruitment [Step 4] and natural killer cell recruitment [Step 4]), as well as high levels of immunosuppressive features (myeloid dendritic cells, Treg cells, infiltration of immune cells into the tumor [Step 5], and recognition of cancer cells by T cells [Step 6]).

Consistently, the 5 mC score showed a significant positive correlation with the infiltration of CD8^+^ T and Treg cells (Figure 3G). In addition, the 5 mC score showed a significant negative correlation with the infiltration of immune cells into the tumor (Step 5) and the infiltration of macrophages M1 and macrophages M2 (Figure 3F).

In summary, cluster 1 and a high 5 mC score predicted a “CD8 T cell-hot” and immune-counterbalanced type tumor (with coexisting immunoactivation and immunosuppression).

### Clustering (according to the 5 mC score) effectively predicted patient response to immunotherapy

**FIGURE 4:**
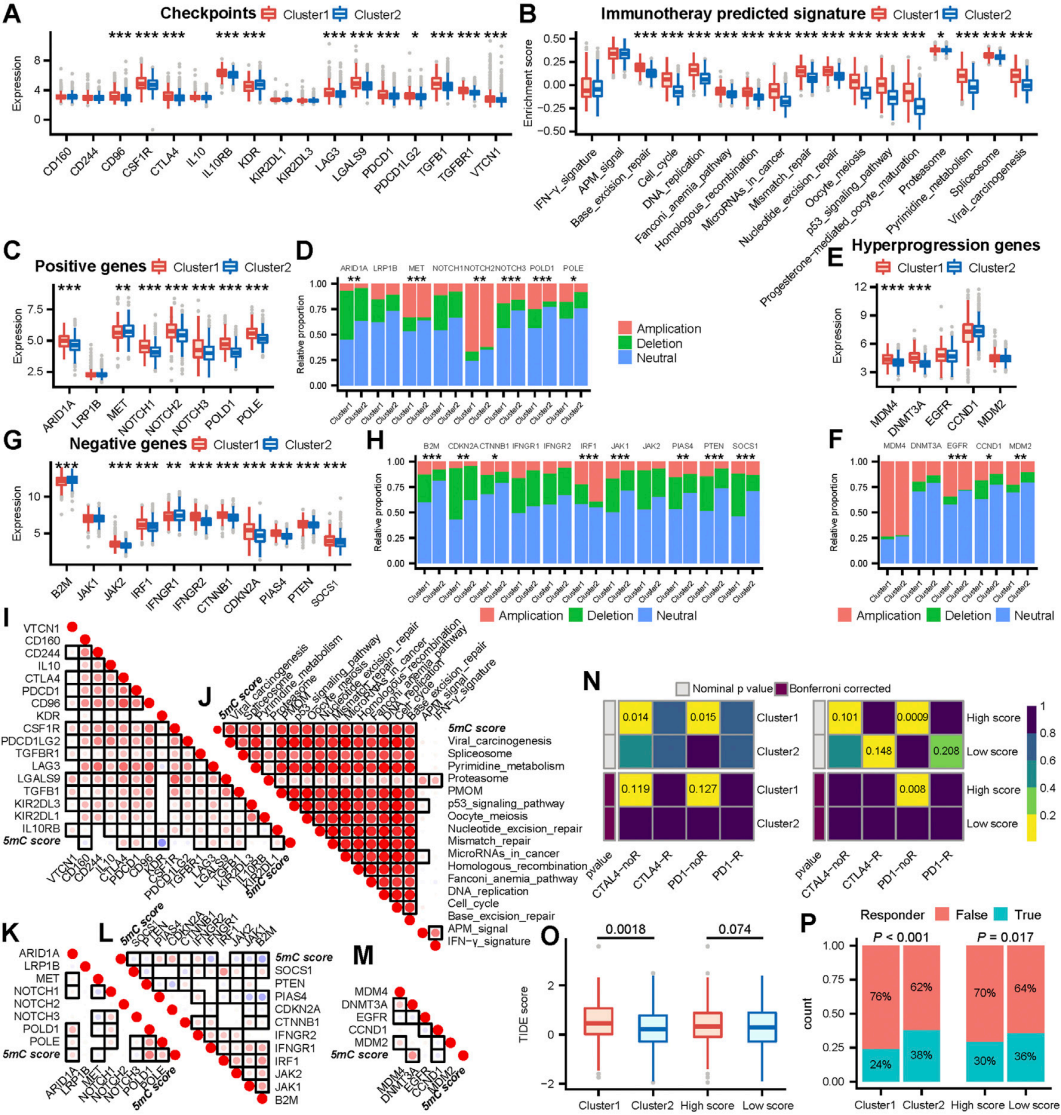
5mC cluster 1 and a high 5 mC score were correlated with a worse ICB response in the meta cohort. **(A)** Differences in the level of immune checkpoint expression between 5 mC clusters. **(B)** Differences in the enrichment scores of positive ICB response-related signatures between 5 mC clusters. **(C)** Differences in the level of positive ICB response-related gene signature expression between 5 mC clusters. **(D)** Differences in copy number alterations of positive ICB response-related gene signatures between 5 mC clusters. **(E)** Differences in the level of ICB hyperprogression-related gene signature expression between 5 mC clusters. **(F)** Differences in copy number alterations of ICB hyperprogression-related gene signatures between 5 mC clusters. **(G)** Differences in the level of negative ICB response-related gene signature expression between 5 mC clusters. **(H)** Differences in copy number alteration of negative ICB response-related gene signatures between 5 mC clusters. **(I)** Correlation between the level of immune checkpoint expression and 5 mC scores. **(J)** Correlation between the enrichment scores of positive ICB response-related signatures and 5 mC scores. **(K)** Correlation between the level of positive ICB response-related gene signature expression and 5 mC scores. **(L)** Correlation between the level of negative ICB response-related gene signature expression and 5 mC scores. **(M)** Correlation between the level of ICB hyperprogression-related gene signature expression and 5 mC scores. For (I)-(M), each point represents the correlation between 5 mC scores and the expression of each gene. The points are color-coded by correlation. The red color indicates a positive correlation, whereas the blue color represents a negative correlation. The light color represents a low correlation and progressively darker colors represent a higher correlation. The black box represents a significant correlation (*p* < 0.05). **(N)** The subclass mapping analysis showed a significant difference in response to anti-PD-1 therapy among 5 mC clusters and score groups. Each cell is color-coded by *p* value according to the color legend. **(O)** The TIDE analysis indicated that patients in cluster 1 had a worse response to ICB treatment (*p* < 0.05). **(P)** The TIDE analysis predicted that patients in cluster 1 and patients with higher scores had a significantly lower proportion of responders to ICB treatment. *p < 0.05, **p < 0.01, ***p < 0.001, PMOM Progesterone−mediated_oocyte_maturation.

The role of clustering (according to the 5 mC score) in mediating the clinical response to treatment with immune checkpoint inhibitors was indirectly confirmed. In this study, we observed that most immune checkpoints, positive ICB response-related genes, and enrichment scores of positive ICB response-related pathway signatures were significant upregulated in cluster 1 (Figures 4A–C). Moreover, we observed that patients in cluster 1 had higher expression levels of negative ICB response-related gene signatures and ICB hyperprogression gene signatures (Figures 4E,G). The positive, negative, and hyperprogression ICB gene signatures showed a significant higher frequency of genomic changes (Figures 4D,F,H).

Next, we analyzed the associations between the 5 mC score and ICB response signatures. The 5 mC score showed significant positive correlations with most immune checkpoints, positive ICB response-related genes, and enrichment scores of positive ICB response-related pathway signatures (Figures 4I–K). However, it also exhibited a significant positive correlation with numerous negative and hyperprogression ICB gene signatures (Figures 4L, M).

These outcomes suggested that patients in cluster1 and those with high 5 mC scores may have worse clinical response to ICB immunotherapy. To validate this inference, we assessed the clinical response of all patients to ICB immunotherapy. Initially, we performed subclass mapping using another cohort of patients with melanoma (n = 47) who received immunotherapy. The submap outcome indicated that remarkable no response inclination for patients in cluster1 (Figure 4N). In cluster 2, there was no clear observation regarding response to ICB. Patients with a high 5 mC score were not sensitive to treatment with programmed cell death 1 (PD1) (Figure 4N).

Next, we used the TIDE algorithm to evaluate potential response to immunotherapy for each patient. Higher TIDE scores indicated less responsiveness to ICB immunotherapy.

We observed that patients in cluster 1 showed significantly higher TIDE scores than those in cluster 2 (Figure 4O). The high 5 mC score group showed an increasing trend in the TIDE score compared with low 5 mC score group, though the difference was not statistically significant (Figure 4P). These findings demonstrated that patients in cluster 1 or patients with a high 5 mC score were potentially insensitive to ICB immunotherapy. These results confirmed the value of 5 mC-based clustering and the 5 mC score.

### 5 mC score predicted classical molecular subtypes and therapeutic opportunities for patients with HCC

**FIGURE 5:**
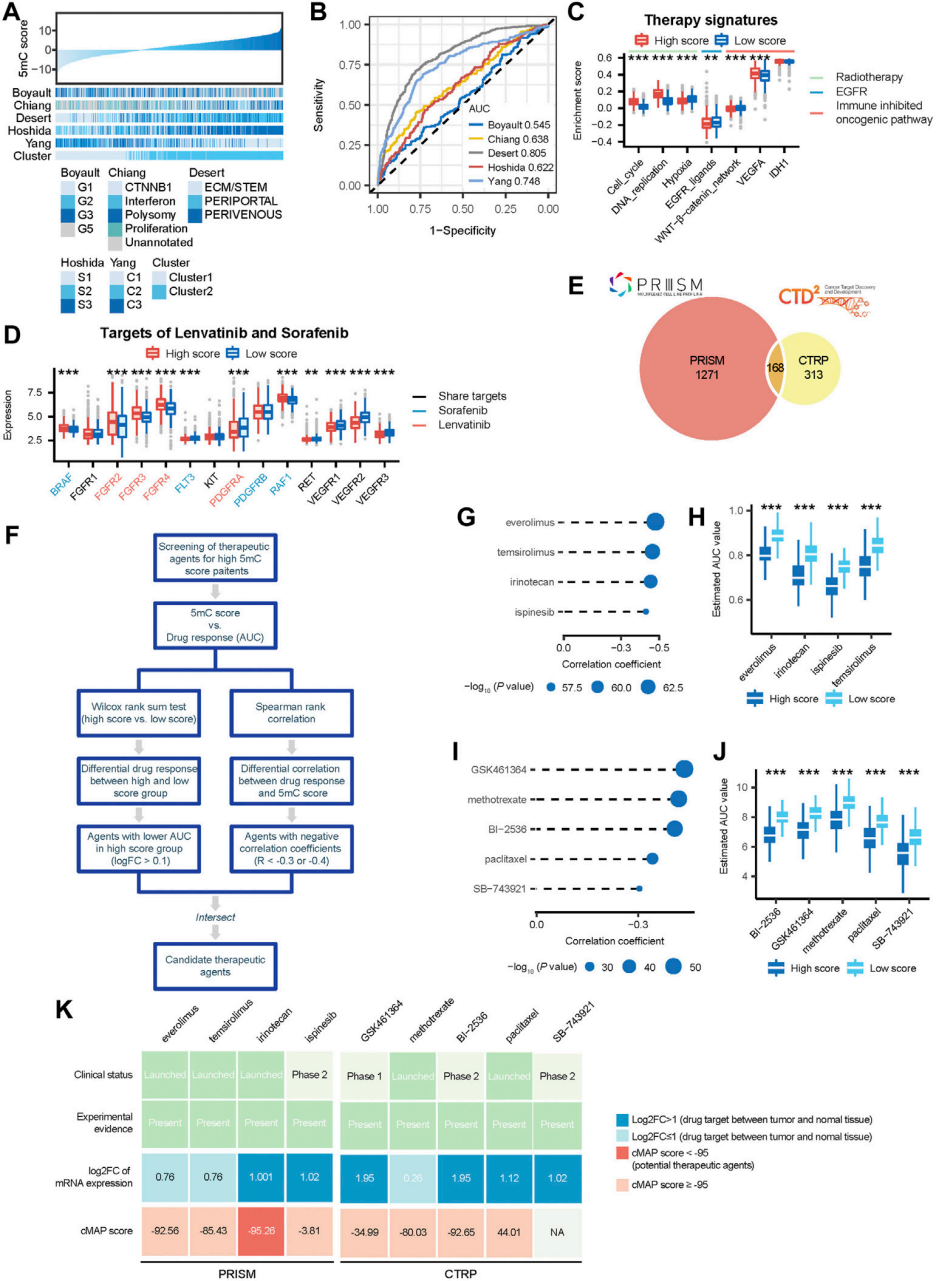
5mC scores accurately predicted classical molecular subtypes and therapeutic opportunities in the meta cohort. **(A)** The association between 5 mC scores and classic molecular subtypes. **(B)** ROC curves showed the accuracy of the 5 mC score in predicting classic molecular subtypes. **(C)** Difference in the enrichment scores of several therapeutic signatures, such as targeted therapy and radiotherapy, between the high and low score groups. **(D)** Comparison of the level of lenvatinib and sorafenib target expression between the high and low score groups. **(E)** A Venn diagram of compounds from the CTRP and PRISM datasets. **(F)** The workflow of identifying potential therapeutic agents. **(G)** Spearman correlation between 5 mC scores and drug response predicted by PRISM datasets. The point size was negatively correlated with the *p* value. **(H)** Comparison of drug response predicted by PRISM datasets between the high and low score groups. **(I)** Spearman correlation between 5 mC scores and drug response predicted by CTRP datasets. Point size was negatively correlated with the *p* value. **(J)** Comparison of drug response predicted by CTRP datasets between the high and low score groups. **(K)** Identification of most promising therapeutic agents for patients with high PPS scores according to the evidence from multiple sources. *p < 0.05, **p < 0.01, ***p < 0.001.

We analyzed the association between 5 mC score (5 mC-based clusters) and five classical molecular subtype classifications (Figure 5A). We observed that 5 mC scores were higher in G2 and G5 proposed by Boyault et al., polysomy suggested by Chiang et al., PERIPORTAL and PERIVENOUS established by Désert et al., S3 suggested by Hoshida et al., and C1 proposed by Yang et al. (Supplementary Figures S4A–E). The ROC curves showed that the 5 mC score predicted classical molecular subtypes established by Désert et al. and Yang et al. with high accuracy (Figure 5B).

Next, we analyzed the association of therapeutic opportunities with high and low 5 mC scores. Our results showed that patients in the high 5 mC score group had significantly higher levels of cell cycle and DNA replication, but significantly lower levels of hypoxia and EGFR ligands (Figure 5C). These findings indicated that patients in the high 5 mC score group may be more sensitive to radiotherapy, while those in the low 5 mC score group may be more sensitive to EGFR targeted therapy. Furthermore, we observed that patients in the high-score group had significantly higher levels of the WNT−β-catenin network, but significantly lower levels of vascular endothelial growth factor A, compared with those in the low-score group (Figure 5C). This evidence indicated the potential involvement of different immunosuppressive mechanisms in the high and low 5 mC score groups. Therefore, targeting these oncogenic pathways may offer promising therapeutic opportunities for patients with HCC. Next, we compared the expression levels of the targets of lenvatinib and sorafenib between the high- and low-score groups. We observed that seven targets of sorafenib (BRAF, FLT3, RAF1, RET, VEGFR1, VEGFR2, and VEGFR3) had significantly different expression in patients with high m5C risk scores (Figure 5D). Most of the targets of sorafenib, except BRAF and RAF1, had lower levels of expression in the high-risk score group (tratio = 5:7). This finding indicated that patients with higher risk scores may be insensitive to sorafenib compared with low-risk patients. We observed that eight targets of lenvatinib (FGFR2, FGFR3, FGFR4, PDGFRA, RET, VEGFR1, VEGFR2, and VEGFR3) had significantly different levels of expression in patients with high m5C risk scores (Figure 5D). Most of the targets of lenvatinib, except FGFR2, FGFR3, and FGFR4, also had a lower level of expression level in the high-risk scores group (ratio = 5:8). Thus, patients with high-risk scores may be less sensitive to sorafenib and lenvatinib compared with low-risk patients.

### Estimation of drug response in patients with HCC

The CTRP and PRISM datasets contain the gene expression profiles and drug sensitivity profiles of hundreds of CCLs. In this study, we used these two datasets to construct a prediction model of drug response. The two datasets shared 168 compounds. After removing duplication, there were 1752 compounds in total (Figure 5E). We excluded compounds with NAs in more than 20% of the samples and cell lines derived from hematopoietic and lymphoid tissue. Finally, 680 CCLs for 354 compounds in the CTRP dataset and 480 CCLs for 1,285 compounds in the PRISM dataset were used for subsequent analyses. The specific screening process is shown in Figure 5F.

Differential drug response analysis was performed between the high 5 mC score group (upper decile) and low 5 mC score group (lower decile) to identify the compounds which showed low estimated AUC values in patients with high 5 mC score. Next, Spearman correlation analysis based on the AUC value and 5 mC score was performed to select compounds with negative correlation coefficients (Spearman’s r: < −0.3 for CTRP or < −0.4 for PRISM). These analyses yielded five CTRP-derived compounds (GSK461364, methotrexate, BI−2536, paclitaxel, and SB−743921) (Figures 5G,H) and four PRISM-derived compounds (everolimus, temsirolimus, irinotecan, and ispinesib) (Figures 5I,J). All these compounds had lower estimated AUC values in the high 5 mC score group versus the low 5 mC score group and a negative correlation with the 5 mC score. Patients with high 5 mC scores showed higher sensitivity to the nine candidate compounds identified versus those with low 5 mC scores. To further support these results, multiple perspective analyses were subsequently conducted to investigate the therapeutic potential of these compounds in HCC.

Firstly, we used cMap analysis to identify compounds which induced gene expression patterns that were oppositional to the HCC-specific expression patterns (i.e., gene expression increased in tumor tissues, but decreased by treatment with certain compounds). The outcomes of the cMap analysis showed scores of irinotecan <−95 (Figure 5K). This suggested that irinotecan has a potential therapeutic effect in HCC.

Secondly, the fold-change in the expression of target genes of candidate drugs between tumor tissues and normal tissues was calculated. A higher fold change value indicated greater potential for a candidate agent in the treatment of HCC.

Thirdly, a comprehensive search was performed in PubMed and DrugTarget to obtain experimental and clinical evidence related to these candidate compounds in the treatment of HCC. The results are presented in Figure 5K. Irinotecan, for which robust *in vitro* and in silico evidence was available, was considered to hold the most promising therapeutic potential for HCC patients with high 5 mC scores.

## Discussion

In recent years, the cumulative evidence has demonstrated an essential role of posttranscriptional mRNA modification in tumor initiation and progression, as well as the prognosis of patients (Wang et al., 2013; Gu et al., 2015; Bauer et al., 2016; Gu L et al., 2020; Gu H et al., 2020; Blanco et al., 2021; Chu et al., 2022). As an ancient and highly conserved RNA modification in all domains of life, the process, distribution, and function of 5 mC in mRNAs have emerged as a new layer of epigenetic regulation (Hu et al., 2021a); however, 5 mC remains partially understood, but few studies have focused on how 5 mC sculpts the TME in HCC. Here, we developed a 5 mC-based clustering system through a comprehensive analysis of a large cohort of patients with HCC. This clustering system can accurately predict prognosis, immune phenotypes, immunotherapy response, classical molecular subtypes, and therapeutic opportunities. Furthermore, we constructed and validated a 5 mC score system to classify patients into these clusters.

Although associations between 5 mC and the TME have been previously observed in some contexts (Jiang 2020; Cong et al., 2021), the mechanisms through which 5 mC influences the TME in HCC have not been elucidated. For this purpose, we used seven independent algorithms to assess the infiltration of immune cells in the TME. We observed that patients in cluster 1 exhibited some antitumor characteristics, such as high levels of CD8^+^ T cell and myeloid dendritic cells, high expression levels of major histocompatibility complex molecules, and the presence of immunostimulators. These may contribute to better survival outcomes for patients in cluster 1. However, our findings showed that patients in cluster 1 had significant worse survival outcome than those in cluster 2. We also observed that patients in cluster 1 exhibited some immunosuppressive characteristics, such as more infiltration of Treg cells and higher expression levels of immune checkpoints than those in cluster 2. These immunoinhibitory factors may contribute to immune escape and lead to worse survival.

Currently, the association between 5 mC and immunotherapy is poorly characterized. In this study, we used two algorithms (TIDE and submap) to assess the response of patients to immunotherapy. We found that patients in cluster 1 and those with high 5 mC scores were non-responsive to immunotherapy. For patients in cluster two or those with low 5 mC scores, there was no clear observation regarding response to immunotherapy. In this study, we analyzed the potential mechanisms through which 5 mC reflects the efficacy of immunotherapy. We observed that patients in cluster 1 had significantly higher levels of immune checkpoints, immunotherapy-positive genes, and predicted signatures. The 5 mC score also showed a significant positive correlation with immune checkpoints and immunotherapy signatures. These results suggested that patients in cluster 1 or those with high 5 mC scores may demonstrate better response to immunotherapy. However, patients in cluster 1 exhibited significantly higher expression levels of immunotherapy-negative genes and higher frequency of genomic alterations. A similar trend was observed for the hyperprogression-related genes. The 5 mC score also showed a significant correlation with the expression levels of immunotherapy-negative genes and hyperprogression genes. These findings may explain the low responsiveness to immunotherapy noted in patients in cluster 1 or those with high 5 mC scores.

Several molecular subtypes have been proposed over the past few years, such as those established by Boyault et al., Chiang et al., Hoshida et al., Désert et al. and Yang et al. However, their clinical application may be limited by several factors, such as the complexity of the sequencing method, high economic burden, and long detection period. In this study, we developed a 5 mC-based classification and a 5 mC score system. The present findings showed that the 5 mC score accurately predicted the classifications proposed by Désert et al. (AUC = 0.805) and Yang et al. (AUC = 0.748). More importantly, the 5 mC score predicted the clinical response to several treatment options, including targeted therapy, radiotherapy, and ICB immunotherapy. A high 5 mC score represented sensitivity to radiotherapy and targeted therapy with lenvatinib. Furthermore, we observed significant differences in immune inhibitory oncogenic pathways between patients in the high and low 5 mC score groups. Thus, targeting these pathways may offer promising treatment options for patients in different 5 mC score groups.

Through a machine learning approach, we identified irinotecan as a potentially promising drug for the treatment of HCC patients with high 5 mC scores. Irinotecan, a topoisomerase I inhibitor, has been widely used as a first-line chemotherapeutic agent in multiple anticancer therapies (Xu et al., 2017). Research has demonstrated that SN38 is the active metabolite of irinotecan in the hepatobiliary tree (Kim et al., 2017), and SN38 led to inhibition of HCC growth *in vitro* (Xu et al., 2017). Unfortunately, the objective response rates recorded following monotherapy with irinotecan were only 0–7% (O'Reilly et al., 2001; Boige et al., 2006). Recent evidence demonstrated that combination of dasatinib and irinotecan/SN38 was able to enhance the anti-HCC efficacy of the treatment (Xu et al., 2017). Therefore, this evidence may provide new directions for the clinical treatment of patients with HCC.

## Data Availability

The datasets presented in this study can be found in online repositories. The names of the repository/repositories and accession number(s) can be found in the article/Supplementary Material.

## Supplementary material

Supplementary Figure S1 Molecular features of 5 mC regulators in the TCGA cohort. **(A)** Mutation landscape of 5 mC regulators. **(B)** The left panel shows the copy number variation frequency of 5 mC regulators. The red nodes represent copy number amplification, while the blue nodes represent copy number deletion. The node size was positively correlated with frequency. The right panel shows the correlation between copy number variation and the level of 5 mC regulator expression. The darker red colors represent a higher correlation and the node size was negatively correlated with the P value. **(C)** The left panel showed the methylation of 5 mC regulators. The red nodes represent higher methylation levels in cancer tissues compared with healthy tissues. The blue nodes represent lower methylation levels in cancer tissues compared with healthy tissues. The right panel shows that the correlation between methylation and level of 5 mC regulator expression. The red color indicates a positive correlation, while the blue color indicates a negative correlation. The node size was negatively correlated with the P value. **(D)** The difference in the level of 5 mC regulator expression between cancer and healthy tissues.

Supplementary Figure S2 The the clinical practice value of 5 mC scores. **(A)** The GO enrichment analysis of DEGs between the 5 mC clusters. **(B)** The KEGG enrichment analysis of DEGs between 5 mC clusters. **(C)** The GO enrichment analysis of DEGs between the high and low 5 mC score groups. **(D)** The KEGG enrichment analysis of DEGs between the high and low 5 mC score groups. **(E)** Calibration curve analysis of 5 mC scores. **(F–H)** The 1-, 3-, and 5-years decision curve analysis of 5 mC scores.

Supplementary Figure S3 Infiltration of immune cells using different algorithms. **(A)** Difference in cancer immunity cycles between clusters 1 and 2. **(B–D)** Difference in immune cells in clusters 1 and 2 assessed by CIBERSOT, EPIC, and xCell. *P < 0.05, **P < 0.01, ***P < 0.001.

Supplementary Figure S4 Association between 5 mC scores and classic molecular subtypes. **(A)** Boyault, **(B)** Chiang, **(C)** Desert, **(D)** Hoshida, and **(E)** Yang. ns, not significant, *P < 0.05, **P < 0.01, ***P < 0.001.
