# Supplementary figures and images for: Role of 5-methylcytosine in determining the prognosis, tumor microenvironment, and applicability of precision medicine in patients with hepatocellular carcinoma
Source: Front Genet. 2022 Sep 16;13:984033. doi: 10.3389/fgene.2022.984033 (PMC9523584; doi:10.3389/fgene.2022.984033)

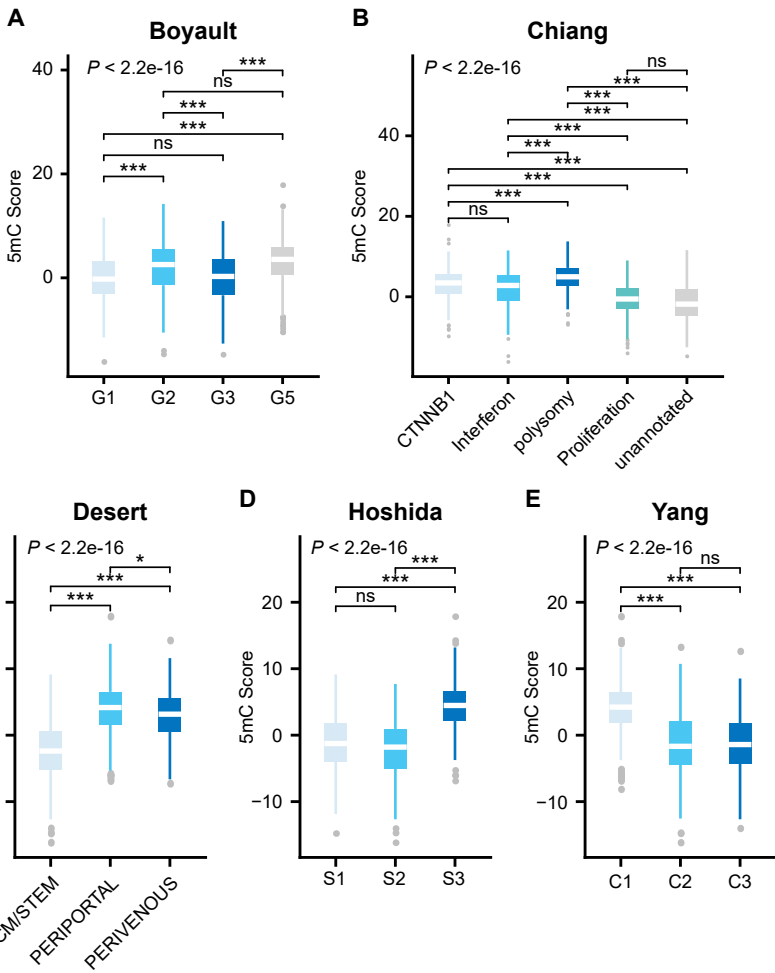

Supplement: Supplementary file 3 [file Image4.PDF]

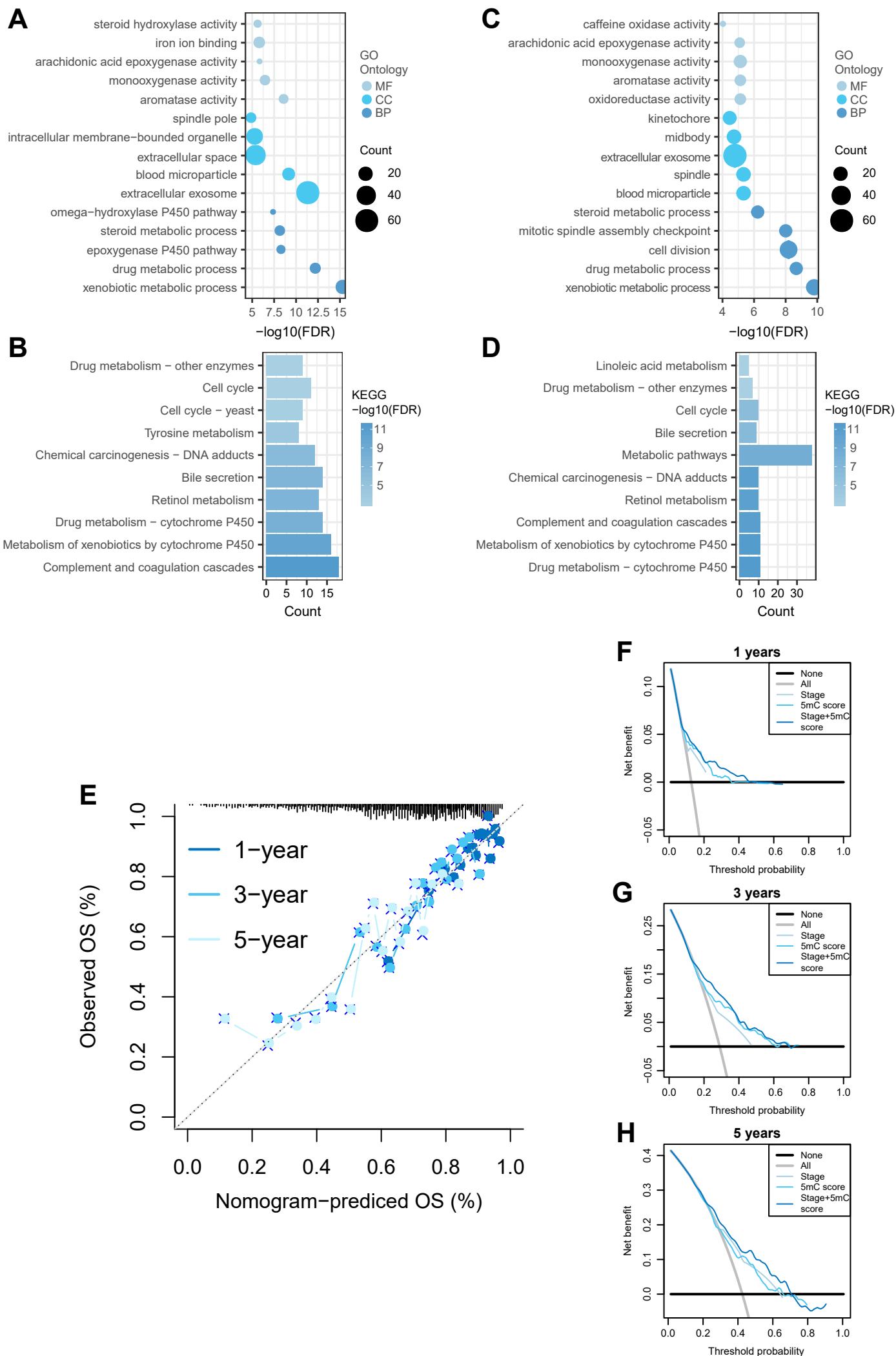

Supplement: Supplementary file 4 [file Image2.PDF]

**A**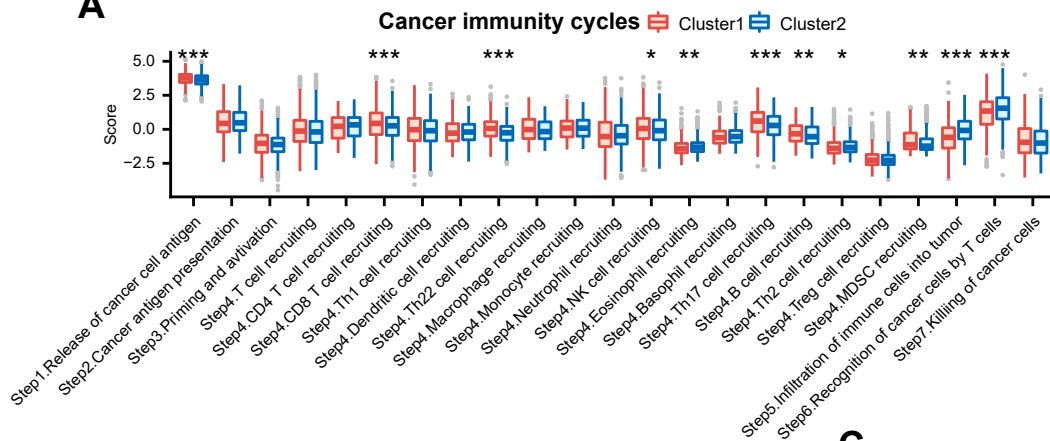**B**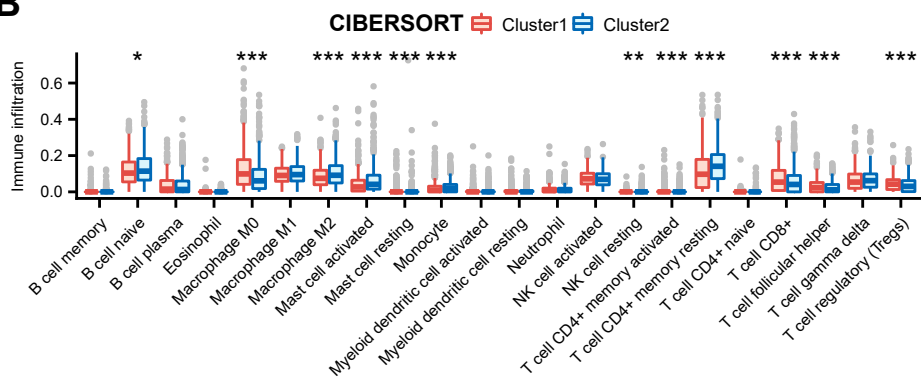**C**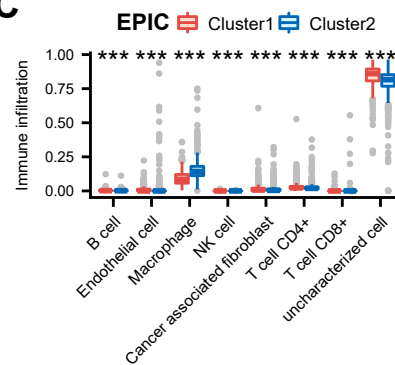**D**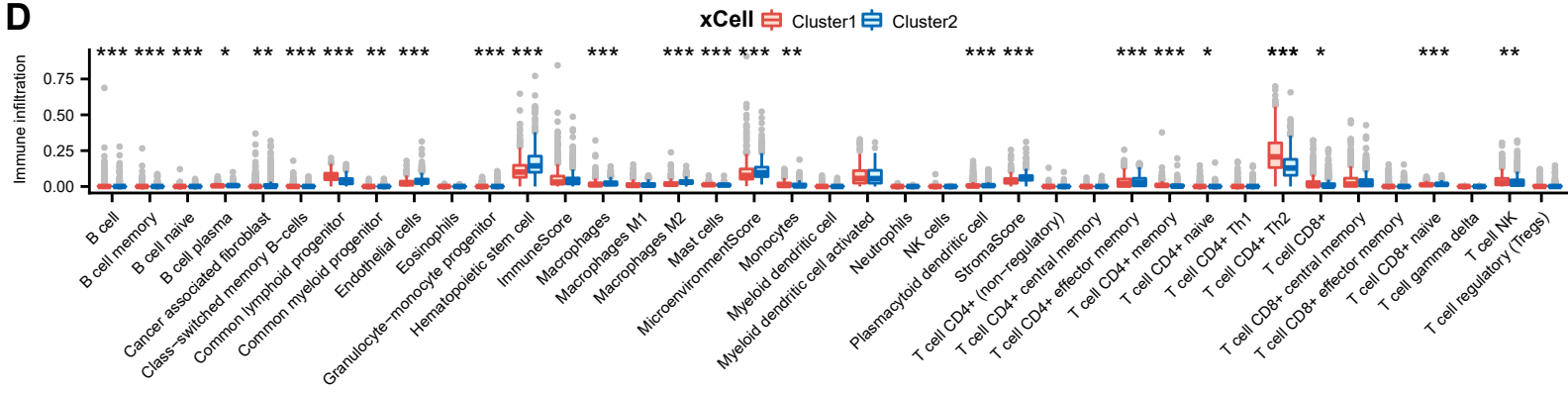

Supplement: Supplementary file 5 [file Image3.PDF]

# A

## Altered in 26 (6.99%) of 372 samples.

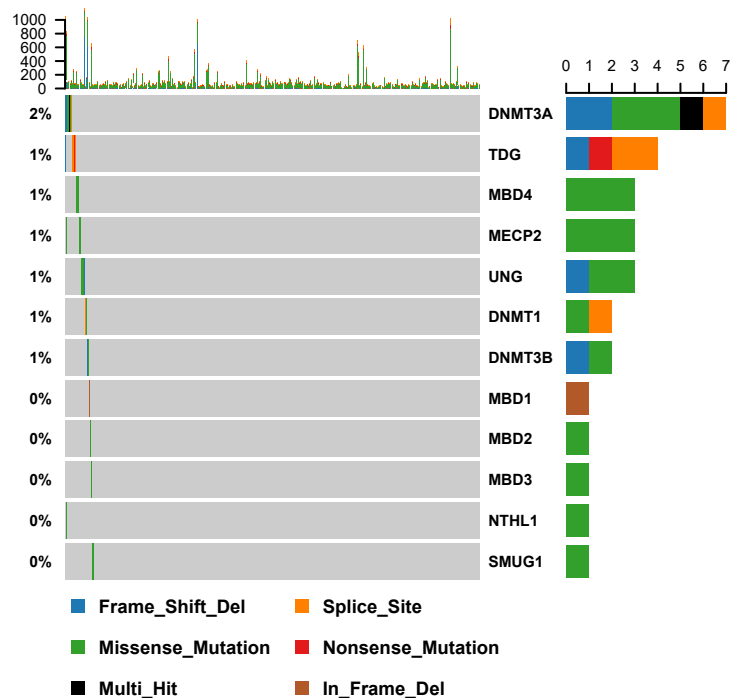

# B

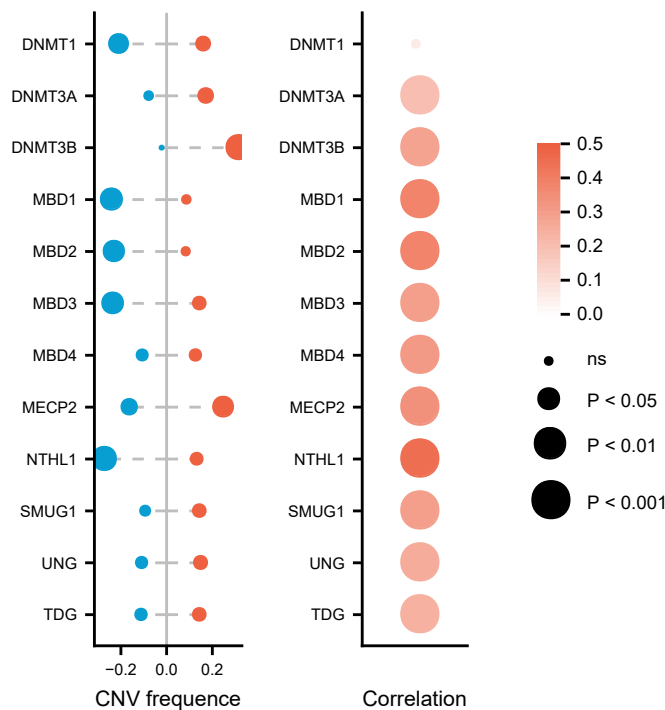

# C

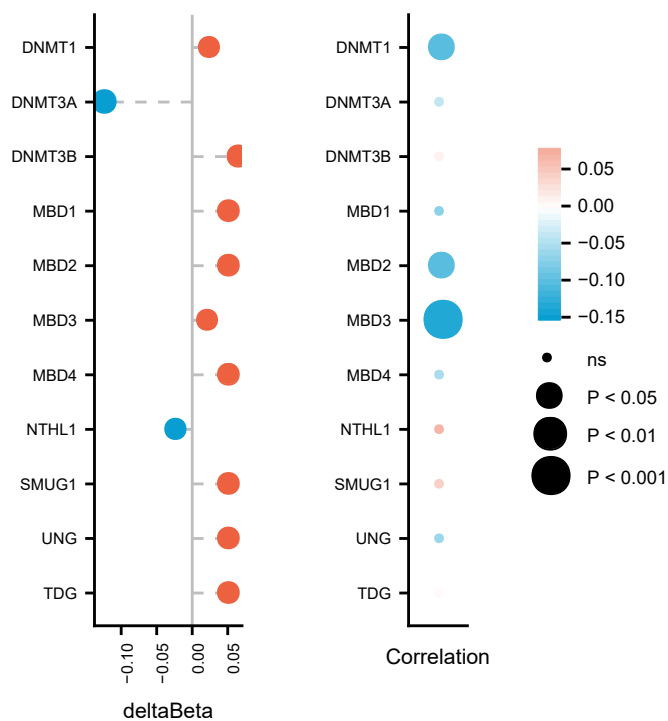

# D

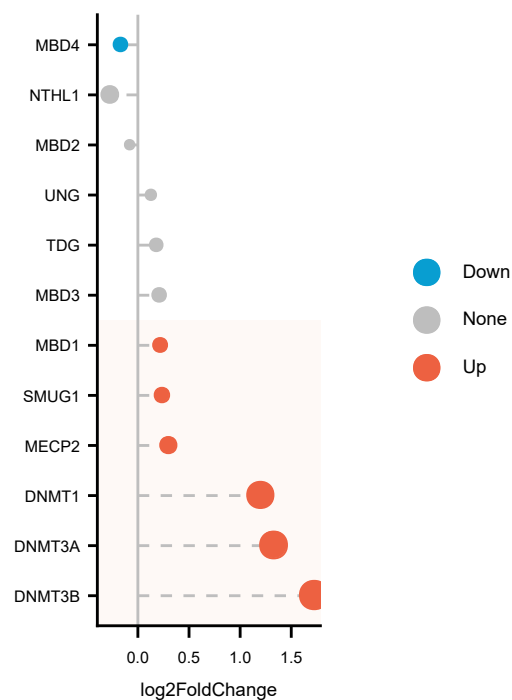

Supplement: Supplementary file 8 [file Image1.PDF]
